# First allergy conference in Qatar: Let's explore allergy with a new lens

**DOI:** 10.5339/qmj.2022.fqac.1

**Published:** 2022-03-30

**Authors:** Maryam Al-Nesf, Tayseer Ibrahim

**Affiliations:** ^1^Allergy and Immunology Division, Department of Medicine, Hamad Medical Corporation, Doha, Qatar E-mail: SPurayil3@hamad.qa

**Keywords:** Qatar allergy, conference, COVID-19 pandemic, virtual meeting

## Abstract

Allergic diseases are common medical conditions that now show an increasing trend globally and contributes to poor quality of life of the affected individual. Allergies can be fatal in a few instances such as anaphylaxis, severe asthma, and hereditary angioedema if the symptoms are not recognized and treated correctly. (1, 2) The first Allergy Conference in Qatar aimed to highlight the burden of allergic diseases in Qatar and globally with a special focus on educating both the physicians and the community. The preparation for this conference over the past three years threw up a few challenges, mainly owing to the coronavirus disease 2019 (COVID-19) pandemic that affected all forms of face-to-face interactions and social gatherings. Therefore, our conference was held virtually on February 05, 2022, which was more than two years from the first planned date. The conference was sponsored by the Hamad Medical Corporation in partnership with the Qatar Allergy and Immunology Society. In our scientific program, the scientific committees selected topics related to 3 major areas in allergy practice (systemic, respiratory, and skin allergy disorders). Abstract submissions were encouraged in areas related to audits, case reports, service development, and clinical and basic research in the field of allergy. Additional topics were included related to the current COVID-19 pandemic and COVID-19 vaccinations. We received 34 abstracts and accepted 28 for poster abstracts. Among them, 6 received approval for oral presentations; and 6 (21.4%) had the term COVID-19 or SARS-CoV-2 in their title, which supported the burden related to COVID-19 in our community and association between this new pandemic and our allergy practice and disorders.

